# Death Be Not Proud: A Commentary on Muslim Acceptance of Death in the Intensive Care Unit

**DOI:** 10.1007/s10943-021-01458-5

**Published:** 2021-11-12

**Authors:** Imran Khan, Ahmed Saad

**Affiliations:** 1 Institute of Population Health Sciences, Barts and The London School of Medicine and Dentistry, Queen Mary University of London, Yvonne Carter Building, 58 Turner Street, London, E1 2AB UK; 2 Ihsan Institute of Islamic Studies, Birmingham, UK

**Keywords:** End-of-life, Islam, Death, Muslims, COVID-19

## Abstract

Technologies used in medicine have meant that treatments can keep people biologically alive but often fail to provide meaningful recovery and quality of life. Many of those from the Islamic faith have relied on these technologies for recovery on religious grounds, even when it may be against clinical advice. This commentary seeks to challenge this notion among many Muslims and suggests there is a psycho-spiritual motivation within the Islamic tradition in not pursuing intensive care treatment that is deemed futile by clinicians. A wish to embrace death in these situations should be expressed to loved ones, and the dying person’s loved ones should be encouraged to embrace death, in order to minimise harm from disagreements between clinical staff and family.

## Introduction

The global pandemic of COVID-19 prompted a discussion around the morality of effective triaging with limited resources during the pandemic (Emanuel et al., 2020). Inevitably, clinicians on the front line prepared themselves to make hard decisions around deciding which patients are prioritised for treatment (Parker & Mirzaali, 2020). In France, ethical support units were set up to provide support to clinicians in making these decisions (Arie, 2020). The units suggested to factor in patient preference in their decision-making, as there are patients who do not wish to be treated should their condition result in an unacceptably low quality of life (Tomlinson, 2020). However, as most serious sufferers of COVID-19 end up on the intensive care unit (ICU), many patients will therefore be unable to communicate any preferences due to the drugs and interventions used to treat them.

Often patients have already expressed their wishes on whether they would like to continue being treated if they lose the ability and cognition to communicate their choices—referred to as advance directives. However, preferences around end-of-life treatment are normally not made or discussed with family, therefore individuals admitted to an ICU in a state of emergency and unexpectedness with often limited awareness, may not have the ability to accept a prognosis of clinical futility. It is therefore up to the family and clinical staff to help in determining what is best for the patient during end-of-life care situations. It is understood the family are in the best position to act as a proxy for the patient’s wishes when the patient is incapacitated. For many Muslim families, there is a willingness to act in accordance with their religious beliefs instead of following clinical advice based on secular bioethical values. In a recent systematic review of preferences and experiences of Muslim patients and their families in Muslim-majority countries for end-of-life care, the authors identify the challenge (in managing end-of-life discussions) of balancing family expectations with the concept of hope in a miracle, and not delaying death when treatment is clinically futile (Abdullah et al., 2020). These differing ethical motivations can conflict, and as a result have led to instances where the end-of-life treatment has been protracted due to the family’s wish to continue active treatment ensuring everything is done to preserve life, and in some cases in direct challenge to the clinicians, who may argue that the harm involved in continuing treatment outweighs the expected benefit (BBC, 2013; Chamsi-Pasha & Albar, 2017). The concept of clinical futility can be complex in the end-of-life context as it is not clearly defined (Truog et al., 1992). What that “harm” involves that compels clinicians to suggest it is worth stopping active treatment, can vary depending on many factors—including limited resource settings where another patient fighting for an intensive care bed has a better chance of survival and improved health outcomes. Also, the scope of what is understood as “harm” in the Islamic context is not clearly defined for end-of-life treatment (Mohiuddin et al., 2020). This is especially relevant during a pandemic where clinicians want to avoid unnecessary use of resources for patients where recovery is unlikely, but also more generally, in assessing whether the “harm” outweighs the expected benefit. However, considering this complexity around the judgement of clinical futility and how it is considered within the Islamic ethico-legal framework, we suggest that following clinical advice can fit within the Islamic paradigm—both from an Islamic legal, and psycho-spiritual, perspectives. It will also have the potential of avoiding any perceived harm from continued treatment (relevant given the importance attached to the dignity of the human body, even after death). As we know there is an emotional and psychological burden on the family following disagreements between the clinical staff and family around end-of-life treatment (Abdullah et al., 2020); avoiding situations that would cause conflict minimises this burden. We are suggesting for Muslims to consider adopting a psycho-spiritual orientation to end-of-life which aims at a death acceptance, while using clinical advice as an anchoring point in deciding when to “let go” and accept death. This anchoring point is generally accepted in Islamic juridical rulings. We give due consideration to the Islamic juridical deliberations towards various aspects of medical futility, e.g. brain death or a persistent vegetative state, however it is not the focus of the article. There are other works such as Mohiuddin et al., (2020), that have analysed these deliberations for each type of situation where continued life support is potentially considered futile by clinicians. For this commentary, we are presenting the reasons for a psycho-spiritual orientation towards death in this context for Muslims.

## The ICU

The modern experience of mortality, as Atul Gwande describes in his 2014 BBC Reith lectures, is an experience that is often highly medicalised and institutionalised. For example, displays of skill, technology and expertise can be often witnessed in the ICU. Typically, this involves a plethora of technology—a mechanical ventilator to replace the action of the lungs when they have failed, an aortic balloon pump when the heart no longer does its job, a dialysis machine to do the work of the kidneys. When you are unconscious and cannot eat, artificial tubing can be surgically inserted into the stomach or intestines to provide nourishment. By design, the ICU provides a high level of clinical care. This level of technology and fantastic ability to heal comes at a price: Firstly, being subject to many of these medical interventions in the ICU can be described as psychological torture (Montgomery et al., 2017). Patients admitted to critical care are often afflicted with post-traumatic stress disorder (PTSD) (Wade et al., 2012), and hallucinations due to the drugs and interventions used to keep them alive. Secondly, due to ICU technology, patients can be kept alive but given no or little chance of recovery by the clinicians who might advise withholding or withdrawing life sustaining treatment despite their very best efforts. Conflicts can arise between the family and the clinical staff when the clinicians assert treatment has no means of bringing about a clinical or “meaningful” improvement and may be causing harm to the patient. However, the family of the patient will not allow withdrawal of treatment as this patient is still alive.

## How do Some Muslims Approach End-of-Life Care in the ICU?

Muslims acknowledge that there is a natural process that will end life. Death is a recurrent theme in the Qur’an and remembrance of death is a foundational spiritual practice (Nasr et al., 2015). There is also an understanding that God can only decide when death occurs (Nasr et al., 2015). Muslims also believe that it is God that heals during sickness: “He who cures me when I am ill” (Haleem, 2007, 26:80). There is an expectation for some Muslims that the interventions used in intensive care are a cure, and a means by which a Muslim must allow healing to take place if one truly believes that God is the only healer. Denying the opportunity for a miracle to occur can be seen as a disbelief in God’s ability to heal (Chamsi-Pasha & Albar, 2017).

This understanding has contributed towards Muslims choosing to prolong treatment clinically judged as futile. In 2013, Mr Khan, a 75-year-old patient who after suffering a cardiac arrest was expected to spend a few days in the ICU following emergency surgery. However, those days turned into months. As time went by it became clear to the clinical team that Mr Khan was not able to survive without ICU treatment. After six months, the clinical team were convinced there was very little chance of Mr Khan making a recovery, and hence proposed to the family (as Mr Khan was unable to communicate) to limit his treatment and allow Mr Khan to die. Mr Khan’s family as Muslims, wanted everything done to preserve life as the ICU machinery was keeping Mr Khan alive, therefore they should leave it in God’s hands to see if Mr Khan recovers. The clinical team objected to continuing treatment as they saw Mr Khan’s chances of recovery very low, and perceived the interventions were causing additional harm to Mr Khan. The clinicians were about to launch a legal case to withdraw life-sustaining treatment, as the family wanted everything possible done to continue active treatment. Following nine months on the ICU, Mr Khan tragically died following a cardiac arrest. There was a ‘do not attempt resuscitation’ order placed on Mr Khan with the family’s agreement, therefore Mr Khan was allowed to die. The family accepted this ending and were satisfied “God’s Will” had been allowed to manifest in the passing of their family member (BBC, 2013).

There have been similar cases where the family have challenged the clinical staff over their decision to withdraw life-sustaining treatment, some of which were referred to the courts to adjudicate (“Family of Muslim man in right-to-life court battle,” 2012; “Religion plea rejected in life-support court case,” 2005).

Cases like these place a burden upon the patient, family, and clinical staff. Does the Islamic tradition necessitate this kind of persistence with end-of-life treatment?

## Islamic Juridical Rulings Towards Withholding or Withdrawing End-of-Life Treatment

It is well established within the Islamic legal tradition that there is no obligation to seek medical treatment near the end of life unless it is likely to lead to clinical benefit. (Padela & Qureshi, 2017). There has also been a recent narrative review of Islamic juridical rulings concerning the question of whether it is ethically justifiable, according to the Islamic ethico-legal framework, to withdraw or withhold life-sustaining treatment (Mohiuddin et al., 2020). The authors reviewed a range of non-binding legal opinions (*fatwās*) from established Islamic jurists and juridical councils and found all the *fatwās* reviewed (*n* = 16) except one, allowed for treatment to be withdrawn or withheld in cases where the clinician prognosticates clinical futility, depressed neurological status, and compounding harms caused from continued treatment. Going back to the case of Mr Khan earlier and for similar cases, this means treatment was not mandatory according to the Islamic legal tradition as it was against the advice of the clinicians who saw Mr Khan as having very little chance of recovery and continued treatment as causing potential harm—to the extent that the clinicians launched a legal case to withdraw treatment. Therefore, Mr Khan’s family should not have insisted on treatment based on *religious grounds*.

### Do clinicians Get it Right?

However, clinicians can make misjudgements in determining futility, and there have been successful challenges to clinical futility which resulted in the patient recovering when the family have insisted on treatment, (“Tafida Raqeeb: Brain-damaged girl in High Court case out of intensive care,” 2020). This can cause confusion for many Muslims in navigating situations like these as to how far they should pursue life-sustaining treatment. In addition to this, establishing when someone has died is difficult to judge. Muslims generally believe death occurs when the soul departs from the body; however, it is debated whether there are physiological manifestations to indicate it (Moosa, 1999). A cartesian mechanistic view of the human body influences most clinical decisions of futility that ignores the existence of a soul, however what does a clinical decision of futility look like on the ICU with the soul considered? This has led to discussions around introducing new categories of death relative to the purpose the death needs to be declared for, e.g. organ donation (Padela & Auda, 2020).

### The Counsel of the Clinician

To summarise the points mentioned from the discussion above: (1) Ambiguity around when to withdraw/withhold treatment considering the limitations of clinician prognostication. (2) Uncertainty from a holistic perspective when the soul has left the body while being actively treated. (3) The consensus of accepting futility as determined by a clinician according to Islamic juridical rulings. Considering these three points, a clinical decision of futility can be deemed an anchoring point in which Muslims can move towards death acceptance. If the appropriate moment to withhold/withdraw treatment is purely judged from a juridical perspective, considering these ambiguities around when the soul has left the body, and the secular bioethical perspective of the clinician when judging what counts as “harm” and “futility”, these can be grounds for further indecision and tension. We suggest that a psycho-spiritual orientation of the patient and family that is rooted in the Islamic tradition can help to navigate situations like these. It is an orientation towards death acceptance when it is religiously acceptable and ethical to do so. Before we describe how this psycho-spiritual orientation is rooted in the Islamic tradition and can help Muslims to find meaning through this approach, there is a different psycho-spiritual orientation that is often taken. Muslims seeking to prolong treatment in hope of a miracle; Is there any problem with this orientation?

## Between Spirit and Law

In many cases involving Muslim patients and family, an approach to navigating end-of-life care situations where the clinicians have indicated there is very little chance of recovery for the patient, has been to hope for a miracle, i.e. the belief in a divine intervention (Abdullah et al., 2020). As already discussed, there have been cases where insistence on continuing life sustaining treatment against clinical advice has led to the recovery of the patient, or as many Muslims would describe as a “miracle.” This sense of hope can be viewed as a form of psycho-spiritual orientation towards end-of-life care. The Islamic tradition recognises there to be a spiritual gain in seeking to prolong treatment for as long as possible as a way of finding meaning and value for those caring for the incapacitated patient. For example, caregivers for patients toward the end of their life have reported a greater sense of responsibility, and experienced becoming closer to God (Abdullah et al., 2020). With respect to the patient, the illness itself can be seen as a purification of sins (Sachedina, 2005), and therefore with this belief a Muslim family could well see that a life is meaningful to sustain despite clinicians wanting to withdraw or withhold treatment and allow death to occur.

Although the above are examples of Muslims finding a spiritual motivation in prolonging life-sustaining treatment, we must ask whether there is any suffering caused by the prolonging of invasive treatment, and if so, should this hope in a miracle continue while there is this suffering? When potential harm is being inflicted from the interventions used to treat the patient, is it acceptable to believe this harm is justified because the patient undergoes purification of their sins? Is it right to burden loved ones with caregiver responsibilities without knowing the effect of any psychological burden on them and whether they can bear it? More generally, what if the collective harm (e.g. potentially on the patient from the continued treatment, burden on the family, and potential conflict with the clinicians) caused to the patient and family in this situation outweighs the perceived value-based gain? There is a principle within the Islamic tradition that mandates not to cause harm or reciprocate harm (Padela & Qureshi, 2017), therefore if greater harm does result from the continuation of futile treatment, this should be avoided based on this principle. In addition to this, approaches that demand the continuation of life-sustaining treatment against clinical advice generally cannot be made on religious grounds, as we have discussed previously. Consequently, it appears that an approach that seeks to prolong life-sustaining treatment against clinical advice, despite having spiritual significance for Muslims, has the potential to lead to discordance with the Islamic juridical rulings if that approach was made on religious grounds.

We therefore propose that the preferred psycho-spiritual orientation rooted in the Islamic tradition that has a greater chance of being concordant with the Islamic juridical rulings, is to not prolong unnecessary medical interventions that go against clinical advice, and to accept death when the clinical team advise the discontinuation of treatment.

## The Soul at Rest

One of the main answers religion provides us with is around making sense of death, to the extent that some would argue death was the main impetus for religions to develop (Jong & Halberstadt, 2016). In the Islamic tradition, a consistent theme in the Qur’an is highlighting the ephemeral nature of the life of this world: “Every person shall taste death” (Haleem, 2007, 3:185), and “what is the life of this world except deceptive enjoyment” (Haleem, 2007, 57:20). Furthermore, in the Islamic tradition, Al-Ghazali—a twelfth century Muslim theologian devoted a volume to “The Remembrance of Death and the Afterlife” in his most famous work “The Ihya Uloom-Al Deen” (“Bringing to Life the Religious Sciences”). In it, he titles a chapter “On the sayings of the caliphs, princes, and righteous men when nearing death”, where he relates stories of righteous figures and their mindset when death approached. He related that when Umar ibn Abdul Aziz (a Muslim ruler held in high esteem by many within the Islamic tradition) became increasingly weighed down by an illness, a physician was summoned:

When he [Physician] looked at Umar and said, “I believe that the man has been poisoned, and I cannot protect him from death.” Umar looked up and said, “Neither can you protect from it the man who has not been poisoned.” Did you feel it, O commander of the Faithful?” the physician asked, and he replied “Yes, I knew it as soon as it entered my belly.” “Accept treatment, O Commander of the Faithful”, he said, “for I fear that your spirit will pass away!” “My Lord is the best destination,” he replied. “By God, if I knew that my cure lay at my earlobe, I would not raise my hand to take it. O Lord God! Allow Umar to choose to meet you!” And no more than a few days later he died. (Al-Ghazali, 1989 pp. 87)

In the exchange between Umar and his physician, it is very clear that Umar has no desire to prolong treatment. His response in refusing the medication the physician is offering seems contrary to attitudes many Muslims have today when facing end-of-life care. Umar’s statement of “By God, if I knew the cure lay at my earlobe, I would not raise my hand to take it” should not be taken literally. This statement is rhetorical, to emphasise Umar’s desire and focused mindset on accepting death and meeting God. In other writings, Al-Ghazali does not deem it compulsory to accept treatment if there is doubt in its efficacy; however, he would deem effective treatments obligatory to accept (Padela, 2013). The Prophetic guidance also makes it clear that Islam is not a “death cult” and rather one should long for life as long as it is good with God, and ask Him to take their life when death is the best thing for them (Prophetic Tradition) (Ali, 2015).

### The Miracle of Mercy

The Quran states: “ward off death from yourselves, if what you say is true” (Haleem, 2007, 3:168); “death will overtake you no matter where you may be, even inside high towers” (Haleem, 2007, 4:78); and, “the Angel of Death put in charge of you will reclaim you, and then you will be brought back to your Lord (Haleem, 2007, 32:11). These and other similar verses describe human beings as powerless in the face of death. Ali ibn Ali Talib, the Prophet Mohammed’s cousin and forth Caliph who narrates the Prophetic saying: “People are asleep, and when they die, they awaken”, informs us that death is actually an awakening to the real existence of the afterlife (Nasr et al., 2015). The psycho-spiritual orientation towards death acceptance can fit into the Muslim’s grand narrative of life and death and can be a source of meaning and realisation of the individual’s ultimate journey back to their Lord. The thirteenth century Muslim theologian Ibn al-Qayyim al-Jawziyya describes the “two wings” that an individual should journey with to their Lord—“awareness of his own faults and recognition of his Lord’s grace” (Al-Jawziyya, 2000, pp. 4). Al-Ghazali also mentions that a person should maintain a favourable opinion of their Lord at the time of death. Al-Ghazali narrates an incident of a man who describes himself as being “immersed in sin” and was praised for hoping for the mercy of his Lord at the time of his death. Al-Ghazali then follows this with the Prophetic tradition in which God says: “I am as my servant thinks of me” (Al-Ghazali, 1989). Ibn al-Qayyim and Al Ghazali are highlighting here that when contemplating one’s own mortality, one should be hopeful in receiving God’s mercy. If this is how they perceive God to be, then they will find it true. From this, it appears the miracle that Muslims ought to be seeking is the miracle of God’s mercy.

Given that Muslims believe that death occurs when the soul departs the body, we understand that an individual may want active treatment against clinical advice through an intuitive or religious belief their loved one can still recover. We are not arguing for this psycho-spiritual orientation of death acceptance to exist in all situations, rather we suggest that it should challenge the view of pursuing active treatment against clinical advice. Muslims have plenty of motivation through their religious tradition to do so.

## Conclusion and Future Directions

Despite Islam’s claim to make death more a part of life, Muslims have found negotiating end-of-life situations challenging. It can be confusing for many Muslims as to when they should decide to withdraw or withhold treatment for themselves or a loved one. The default spiritual outlook for Muslims has been to seek the means for God’s intervention through the machinery of the ICU, and therefore doing everything in their power to continue treatment.

We have shown that Islamic juridical rulings permit treatment that the clinician determines as ineffective to be withdrawn or even refused. We have also shown that the Islamic tradition encourages a spiritual orientation towards death acceptance. It is therefore within the Islamic tradition for the clinical decision to be an anchoring point to accept the outcome of death; hence it ought to be the default action rather than seeking active treatment in search of a miracle. With the insistence on prolonging active treatment, it suggests that God can only act through the intensive care apparatus, and if one accepts withdrawal of active care due to perceived futility, this is blocking the means for God to cure the individual thus lacking faith in God. In this scenario, God is restricted to intervene through the machinery of the ICU.

We are also attempting to highlight that approaching death can be a pathway for the dedicated believer to find meaning. Rather than fighting the grip of death, Islam can see it as a merciful embrace. Death in the ICU viewed this way can be a means to achieve a spiritual death in a world focused on the physical. Being “religious” in this situation does not mean praying for the miracle in the technology, but we have argued it can be “religious” to embrace the inevitable and accept mercy over assimilation by the ICU machinery. When to make that judgement to let go can be anchored around clinical judgement. The Islamic tradition is clear in that those who love to meet God, God would love to meet them (Sahih Muslim: Book 35, Hadith 6490).

Though the process of dying and death in the ICU is a very sensitive time, there is a need for empirical research to explore attitudes of Muslim patients and families during this process. Such research will be valuable in understanding the motivations of Muslims behind their decision-making in these circumstances in order to inform clinical teams and families come to the best decisions.
